# Hydrocephalus Complicating Breech Presentation

**Published:** 1886-12

**Authors:** Edmund J. Doering

**Affiliations:** President Chicago Medical Society; Chicago, Illinois


### HYDROCEPHALUS COMPLICATING A BREECH PRESENTATION.

*By Edmund J. Doering, M. D., President Chicago Medical Society.*

(For Daniel's Texas Medical Journal.)

THE writer was very much interested in the article on "Craniotomy," by Dr. Peyton Turner, in the October number of DANIEL'S MEDICAL JOURNAL. The Doctor managed his case admirably, and in accordance with the teachings of the recognized authorities. It seems strange that after having saved the life of the mother, for which he should have received unstinted praise, an attempt should have been made to find a criminal indictment against him for having done his duty. It simply illustrates that there is absolutely no limit to the amouut of ingratitude and ignorance of which a human being may be possessed. Dr. Turner's case leads me to place on record a similar one which occurred in my practice about two years ago.

On the morning of the 10th day of October, 1884, I was called to attend Mrs. Z.——, age 24, whom I had attended a year previous in her first confinement, delivering her of a strong, healthy child. Mrs. Z.—— informed me that she had had more or less pain for 24 hours preceding my arrival. On examination I found the os sufficiently dilated to diagnose a breech presentation. The pains continued frequent and severe, the os dilated completely in a few hours, and the breech descended into the cavity of the pelvis, where it remained stationary and apparently firmly impacted. After waiting patiently for two hours and no change taking place, I sent for my friend, Dr. S., to assist me in giving an anæsthetic, and with some difficulty delivered the breech with the forceps. Laying aside the forceps, I extracted the child as far as the neck, and here all my efforts to deliver the head failed. Dr. S. then changed places with me, but with the same result. From the impossibility of applying the forceps to the after-coming head, we concluded that we had a hydrocephalic head to deal with, and after some delay in getting the necessary instruments, with the assistance of Dr. C., the head was perforated and extracted with a blunt hook. The head measured about twice the size of an average head. The mother did remarkably well and recovered without one unfavorable symptom.

The treatment consisted of antiseptic injections and the internal administration of ergot and quinine.

In June of the present year I delivered Mrs. Z. of a healthy girl, a face presentation, but requiring no interference. It may be of interest to quote here a few extracts from the works of different authorities: Lusk, 3d edition, 1885, says: "Congenital hydrocephalus of sufficiently marked development to constitute an impediment to parturition, is comparatively rare. occurring, according to the statistics of Madame LaChapelle, only 15 times in 43,545 deliveries. In case of a breech presentation the diagnosis, which is then more difficult, must chiefly rest upon the detection, at the fundus, of a tumor larger than the normal fœtal cranium. The previous occurrence of hydrocephalus in the same subject and feeble fœtal movements may, in this instance, slightly facilitate the task of the diagnostician."

Cazeaux & Tarnier (8th American edition, 1886,) wrote as follows: "Hydrocephalus becomes a more serious matter when the breech presents, inasmuch as the true nature of the case is liable to escape detection. Again, supposing the diagnosis made out, perforation of the cranium is performed with difficulty, on account of its being accessible only by its base. Though it is often possible to pass the instrument through the arch of the palate, I would prefer to introduce the blunt hook into the orbit and enter the cranium through the optic foramen." (This was the method followed in Mrs. Z.'s case.)

In the 2d edition of Playfair he writes: "The diagnosis of intra-uterine hydrocephalus is by no means so easy as the description in obstetric works would lead us to believe. As a matter of fact, the true nature of the case is comparatively rarely discovered before delivery. In one out of five cases of hydrocephalus the fœtus presents by the breech. The diagnosis is then still more difficult, for the labor progresses easily until the shoulders are born, when the head is completely arrested, and refuses to pass with any amount of traction that is brought to bear on it. Even the most careful examination may not now enable us to make out the cause of the delay."

From the above extracts we learn then that hydrocephalus, complicated by breech presentation, occurs about once in 14,000 deliveries, and that the diagnosis is very difficult to make.

